# Wenxin Granule Ameliorates Hypoxia/Reoxygenation-Induced Oxidative Stress in Mitochondria via the PKC-*δ*/NOX2/ROS Pathway in H9c2 Cells

**DOI:** 10.1155/2020/3245483

**Published:** 2020-05-20

**Authors:** Qihui Jin, Yanhong Jiang, Lizhong Fu, Yanqiu Zheng, Yuxia Ding, Qian Liu

**Affiliations:** ^1^Department of Geriatric Medicine of the Second Affiliated Hospital, Zhejiang University School of Medicine, Hangzhou 310009, China; ^2^College of Pharmaceutical Science, Zhejiang Chinese Medical University, Hangzhou 311402, China

## Abstract

Myocardial infarction and following reperfusion therapy-induced myocardial ischemia/reperfusion (I/R) injury have been recognized as an important subject of cardiovascular disease with high mortality. As the antiarrhythmic agent, Wenxin Granule (WXG) is widely used to arrhythmia and heart failure. In our pilot study, we found the antioxidative potential of WXG in the treatment of myocardial I/R. This study is aimed at investigating whether WXG could treat cardiomyocyte hypoxia/reoxygenation (H/R) injury by inhibiting oxidative stress in mitochondria. The H9c2 cardiomyocyte cell line was subject to H/R stimuli to mimic I/R injury *in vitro*. WXG was added to the culture medium 24 h before H/R exposing as pretreatment. Protein kinase C-*δ* (PKC-*δ*) inhibitor rottlerin or PKC-*δ* lentivirus vectors were conducted on H9c2 cells to downregulate or overexpress PKC-*δ* protein. Then, the cell viability, oxidative stress levels, intracellular and mitochondrial ROS levels, mitochondrial function, and apoptosis index were analyzed. In addition, PKC-*δ* protein expression in each group was verified by western blot analysis. Compared with the control group, the PKC-*δ* protein level was significantly increased in the H/R group, which was remarkably improved by WXG or rottlerin. PKC-*δ* lentivirus vector-mediated PKC-*δ* overexpression was not reduced by WXG. WXG significantly improved H/R-induced cell injury, lower levels of SOD and GSH/GSSG ratio, higher levels of MDA, intracellular and mitochondrial ROS content, mitochondrial membrane potential and ATP loss, mitochondrial permeability transition pore opening, NOX2 activation, cytochrome C release, Bax/Bcl-2 ratio and cleaved caspase-3 increasing, and cell apoptosis. Similar findings were obtained from rottlerin treatment. However, the protective effects of WXG were abolished by PKC-*δ* overexpression, indicating that PKC-*δ* was a potential target of WXG treatment. Our findings demonstrated a novel mechanism by which WXG attenuated oxidative stress and mitochondrial dysfunction of H9c2 cells induced by H/R stimulation via inhibitory regulation of PKC-*δ*/NOX2/ROS signaling.

## 1. Introduction

Timely revascularization of occluded vessels in acute myocardial infarction is a common therapy for rescue ischemic myocardium [1]. When oxygen redelivered to hypoxia myocardium, the cardiac ischemia/reperfusion (I/R) injury happens [2]. Cardiac I/R causes paradoxical cardiomyocyte dysfunction, including cardiomyocyte metabolism disorder and cell damage, which contributes to high morbidity and mortality in patients [3]. Bedsides, therapies to suppress I/R injury largely focused on its initiation and regulation mechanisms [4]. Oxidative stress, mitochondrial dysfunction, inflammation, and cell death-induced tissue alterations are considered of great importance in I/R injury development [5, 6]. However, the exact mechanisms of I/R injury remain not fully known.

As the powerhouse of myocardiocytes, mitochondria are considered key participants in myocardial I/R injury [7]. The concept that oxidative stress and calcium-induced mitochondrial dysfunction drives cellular damage is addressed both *in vivo* I/R and *in vitro* hypoxia/reoxygenation (H/R) injury [8, 9]. Reactive oxygen species (ROS) is the main source of oxidative stress in homeostasis disorders when its production exceeds the available antioxidant defense systems [10]. Although low to modest concentration of ROS serves paramount roles in routine physiological functions, uncontrolled ROS generation may arise more oxidative stress and spiral in a cycle of inflammation and oxidative injury, including heart and cardiovascular system diseases [11]. ROS generation in cytosol and mitochondria is considered vital in determining the severity of myocardial damage [9, 12]. Besides the amount of ROS, the site at which ROS are generated should not be neglected either [13].

Nicotinamide adenine dinucleotide phosphate oxidases (NADPH oxidases, NOXs), as a significant intracellular enzymatic source of ROS, comprise of seven members: NOX1, 2, 3, 4, and 5 and Duox1 and Duox2 [14, 15]. Most NOXs are transmembrane complexes with electron-transferring ability to produce ROS [16]. Among them, NOX2 and NOX4 are quite abundant in cardiomyocytes involved in myocardial I/R injury [17]. Researchers have demonstrated that NOX2 occupies the main role in I/R injury-induced ROS generation than NOX4 [18]. Protein kinase C, especially the *δ* isoform (PKC-*δ*) involved pathway, is believed to trigger NOX2 activation [19–21]. The activation of NOX2 starts from the membrane translocation of phosphorylated p47phox [22]. The conformational change of phosphorylated p47phox is facilitated to form a complex with transmembrane subunit p22phox [23], which becomes a new target for antioxidants.

Wenxin Granule (WXG) is recorded in Pharmacopeia of the People's Republic of China (the 2015^th^ edition) as a cardiac arrhythmia therapeutic agent. The formula of WXG consists of Dangshen (*Codonopsis Radix*), Huangjing (*Polygonati Rhizoma*), Sanqi (*Notoginseng Radix Et Rhizoma*), Hupo (Ambrum), and Gansong (*Nardostachyos Radix Et Rhizoma*) [24, 25]. It has been widely used in the treatment of cardiac inflammation, cardiac hypertrophy [26], cardiac arrhythmias [27], atrial premature beats, ventricular premature beats, atrial fibrillation [24], heart failure [28], myocardial oxidative stress [29], and apoptosis [30]. Studies also confirmed the myocardial infarction protection effect of WXG [31]. However, the oxidative stress-related pathways predicted to be targeted by WXG in I/R-induced cardiomyocyte injury are still unclear.

For these, in order to study the effects of WXG on cardiomyocyte I/R injury, we used an H9c2 cell H/R model. In the present study, we aimed to elucidate the connection among oxidative stress-induced PKC-*δ* overproduction, NOX2 activation, and ROS outburst. Furthermore, we focused on the WXG regulation of PKC-*δ* expression, NOX2 activation, ROS production, and mitochondrial function in H/R-induced H9c2 cells. On the basis of this, we hypothesized that WXG ameliorated mitochondrial oxidative stress injury during H/R via the PKC-*δ*/NOX2/ROS signaling pathway.

## 2. Materials and Methods

### 2.1. Drugs and Reagents

WXG was provided by Buchang Company (Xi'an, China). The powdered WXG compound was dissolved in saline solution at a concentration of 5 mg/mL before use. Polybrene, puromycin, rottlerin (Rott), 2′,7′-dichlorodihydrofluorescein diacetate (DCFH-DA), and JC-1 were purchased from Sigma-Aldrich (St. Louis, MO, USA). Dulbecco's modified Eagle's medium (DMEM) and fetal bovine serum (FBS) were purchased from GIBCO (Grand Island, NY, USA). Cell Counting Kit-8 (CCK-8) was purchased from Dojindo (Kyushu, Japan). The lactate dehydrogenase (LDH) assay kit, superoxide dismutase (SOD) assay kit, and malondialdehyde (MDA) assay kit were obtained from Jiancheng Bioengineering Institute (Nanjing, China). The BCA protein assay kit, cytosol and membrane protein extraction kit, GSH and GSSG assay kit, ATP detection kit, and DAPI staining reagent were purchased from Beyotime (Shanghai, PR China). Dihydroethidium (DHE) and the MitoTracker Red CMXRos kit were obtained from Invitrogen (Grand Island, NY, USA). MitoSOX Red Mitochondrial Superoxide Indicator and Calcein-AM (calcein) were obtained from Life Technologies (Grand Island, NY, USA). Terminal deoxynucleotidyl transferase-mediated dUTP nick end labeling (TUNEL) was obtained from Roche Diagnostics (Indianapolis, IN, USA). PKC delta antibody, NOX2/gp91phox antibody, and cytochrome C antibody were obtained from Abcam (Cambridge, MA, USA). Na/K ATPase antibody, P47phox antibody, Bax antibody, Bcl-2 antibody, and cleaved caspase-3 antibody were obtained from Cell Signaling Technology (Beverly, MA, USA). *β*-Actin antibody was bought from Beyotime.

### 2.2. Cell Culture

The H9c2 rat embryonic cardiomyocyte cell line was a gift from Dr. Ling Zhang (Zhejiang University, Zhejiang, China). The cells were cultured in DMEM supplemented with 10% (*v*/*v*) FBS in a humidified incubator (95% air, 5% CO_2_, 37°C).

### 2.3. Lentivirus Construction and Infection

Lentivirus particles carrying PKC-*δ* (Lv-PKC-*δ*) and the empty vectors (Lv-CON) were constructed by Keqing (Hangzhou, PR China). The lentiviral vectors were transfected into H9c2 cells at a multiplicity of infection (MOI) of 10 in the presence of 5 *μ*g/mL polybrene. The growth medium was replaced 24 h after infection. 72 h later, H9c2 cells were screened with 2.0 *μ*g/mL puromycin and cultured in a humidified incubator (95% air, 5% CO_2_, 37°C).

### 2.4. Cell Grouping and Treatment

To verify the effect of WXG on I/R injury *in vitro*, the I/R injury model was mimicked by subjected H9c2 cells with 16 h of hypoxia in the glucose-free medium followed by 2 h of reoxygenation stimulus according to our previous study. H9c2 cells were divided into 4 groups as follows: (1) the control group, H9c2 cells were kept in normal incubator; (2) the H/R group, H9c2 cells subjected to 16 h of hypoxia (O_2_ : N_2_ : CO_2_, 1 : 94 : 5) followed by 2 h of reoxygenation; (3) the H/R+WXG group, H9c2 cells were pretreated with WXG (5 mg/mL) for 24 h prior to and during hypoxia treatment; and (4) the H/R+Rott group, H9c2 cells were stimulated with 5 *μ*M rottlerin for 1 h before H/R treatment.

To demonstrate the correlation between WXG and the PKC-*δ*/NOX2/ROS signaling pathway, H9c2 cells were divided into 6 groups as follows: (1) the Lv-CON group, H9c2 cells were transfected with empty vectors; (2) the Lv-PKC-*δ* group, H9c2 cells were transfected with PKC-*δ* lentivirus particles; (3) the H/R+Lv-CON group, empty vector H9c2 cells subjected to 16 h of hypoxia (O_2_ : N_2_ : CO_2_, 1 : 94 : 5) followed by 2 h of reoxygenation; (4) the H/R+Lv-PKC-*δ* group, PKC-*δ* overexpression H9c2 cells subjected to 16 h of hypoxia (O_2_ : N_2_ : CO_2_, 1 : 94 : 5) followed by 2 h of reoxygenation; (5) the H/R+WXG+Lv-CON group, empty vector H9c2 cells were pretreated with WXG (5 mg/mL) for 24 h prior to and during hypoxia treatment; and (6) the H/R+WXG+Lv-PKC-*δ* group, PKC-*δ* overexpression of H9c2 cells were pretreated with WXG (5 mg/mL) for 24 h prior to and during hypoxia treatment.

### 2.5. Cell Viability Assay

H9c2 cells were grown on 96-well plates at 5 × 10^3^ cells/well 12 h before use. After various treatments described above, CCK-8 assay was used to evaluate the cell viability. Briefly, 10 *μ*L CCK-8 was added to each well and incubated for 2 h. At last, the absorbance of each well was detected at 450 nm using a microplate reader. The replicate size was 6 for each group, and all groups were compared to the control group. The release of LDH into the cell culture medium as an indicator of membrane damage was quantified according to the manufacturer's instructions at a wavelength of 440 nm.

### 2.6. Measurement of SOD Activity, MDA Level, and GSH/GSSG Ratio

To investigate the antioxidant ability of WXG, H9c2 cells were seeded into 6-well plates and treated as described above. The culture medium and H9c2 cells were collected separately. The culture medium was used for measuring SOD activity. H9c2 cells were homogenated after washing with cold PBS, and the supernatants were used for measuring the MDA level and the GSH/GSSG ratio by commercial assay kits according to the manufacturer's instructions, respectively. Levels were standardized using total cellular protein determined by BCA assay.

### 2.7. Intracellular ROS and Mitochondrial ROS Detection

Intracellular ROS levels were determined by DHE and DCFH-DA staining. The pretreated H9c2 cells were incubated with 10 *μ*M DHE solution [32] at 37°C for 30 min without light. Then, DHE was removed by washing with PBS twice. The cells were imaged on a fluorescent microscope camera (Olympus IX71, Olympus Corporation, Tokyo, Japan). And the fluorescent intensity of DHE was analyzed with Image J software (National Institutes of Health, Bethesda, MD, USA). For DCFH-DA staining, after treated with NOX2 inhibitor peptide (GP91ds-tat, 5 *μ*M [33]), scramble GP91ds-tat (scrGP91ds-tat, 5 *μ*M), NAD(P)H oxidase inhibitor (apocynin, 0.3 mM [34]), and mitochondrial ROS scavenger (Mito-TEMPO, 10 *μ*M [33]), H9c2 cells were incubated with 10 *μ*M DCFH-DA [35] at 37°C for 30 min without light. Then, DCF-loaded cells were washed and analyzed by flow cytometry (wavelength 488/538 nm).

For mitochondrial ROS detection, H9c2 cells were loaded with MitoSOX (5 *μ*M [36, 37]) at 37°C for 30 min followed by washing twice with HBSS buffer containing Ca^2+^/Mg^2+^. Subsequently, cells were aliquoted at a density of 5‐10 × 10^6^ cells in a sterile FACS tube and analyzed by flow cytometry (emission wavelength 580 nm).

### 2.8. Mitochondrial Membrane Potential Measurement

Changes of the mitochondrial membrane potential (*ΔѰ*m) were detected using the fluorescent probe JC-1 according to the user manual. JC-1 is a cationic dye that concentrates in the mitochondrial matrix following membrane potential. JC-1 accumulates in the mitochondria in healthy cells, forming J-aggregates and emitting red fluorescence. However, upon cell injury, the *ΔѰ*m is low, and the J-monomers generate green fluorescence. JC-1 (2 *μ*M [38]) was added to individual groups and incubated for 20 min in the dark. After washing twice with PBS, H9c2 cells were observed and the images were captured under a fluorescence microscope Olympus IX70. The mean intensities were analyzed with Image J software. The relative proportion of red and green fluorescence intensity was used to demonstrate the degree of mitochondrial depolarization.

### 2.9. ATP Level Measurement

The intercellular ATP was determined by an ATP detection kit according to the manufacturer's instructions. Briefly, after the indicated treatments, H9c2 cells were washed and homogenated, and the supernatants were collected to measure ATP and total protein content.

### 2.10. Mitochondrial Permeability Transition Pore Opening Assay

Opening of mitochondrial permeability transition pore (mPTP) was examined via a calcein-loading/CoCl_2_-quenching system, a technique enabling visualization of the open/closed status of mPTP. Briefly, H9c2 cells were loaded with 1 *μ*M calcein [39] and 2 mM cobalt chloride (CoCl_2_) for 20 min in darkness. CoCl_2_ eliminated all calcein fluorescence in the cytoplasmic so that only the mitochondria had green fluorescence. After washing away excessive dye and quenching reagent, H9c2 cells were imaged using a confocal laser scanning microscope (LSM 880, Carl Zeiss Meditec, Jena, Germany). The mean intensities were analyzed with Image J software. The changes of green fluorescence intensity in the mitochondria were index of mPTP opening.

### 2.11. Immunofluorescence Staining

H9c2 cells were fixed with 4% formaldehyde and washed with PBS, then permeabilized with 0.2% Triton X. Mitochondria were stained with a MitoTracker Red CMXRos kit. After being blocked with 5% bovine serum albumin, cells were incubated with primary antibodies against cytochrome C and with the corresponding secondary antibodies, and the nuclei were counterstained with DAPI. Six fields were captured for each well using an LSM 880 confocal laser scanning microscope (original magnification, 630x).

### 2.12. TUNEL Assay

Apoptosis was assessed using a commercially available TUNEL assay kit. Briefly, fixed H9c2 cells were washed twice with cold PBS and stained with DAPI and TUNEL following the manufacturer's instructions. After washing, fluorescent images were captured using an Olympus IX70 fluorescence microscope at 200x magnification and analyzed using Image J software. The total number of cells (DAPI positive, blue) and apoptotic cells (TUNEL positive, green) was counted in six random fields for each group. The level of cell apoptosis was evaluated as the ratio of apoptotic cells/total number of cells.

### 2.13. Western Blotting Analysis

To detect the protein expression of PKC-*δ*, p47phox, gp91phox, cytochrome C, Bax, Bcl-2, and cleaved caspase-3, western blotting was carried out. After corresponding treatments, H9c2 cells' total proteins were harvested by ice-cold RIPA lysis buffer with 1 mM phenylmethylsulfonyl fluoride (PMSF). The lysate supernatant was obtained by centrifuging at 10,000 rpm for 10 min at 4°C. Protein concentrations were measured via the BCA Protein Quantification Kit according to the manufacturer's protocol. To detect the translocation of p47phox, the cytosol and membrane proteins were extracted using the commercial kit according to the manufacturer's instruction. Equal amounts of protein samples were transferred to PVDF membranes (Bio-Rad, U.S.) after separated by SDS-PAGE gels. *β*-Actin was used as an internal control of the cell lysates and cytosolic fraction. Na/K ATPase was used as an internal control of the membrane fraction. The corresponding membranes were blocked for 1.5 h and then blotted with specific primary antibodies (1 : 1000) overnight at 4°C. After washing three times with TBST, the membranes were incubated with secondary antibodies (1 : 5000) for 2 h at room temperature. Then, the bands were visualized with an enhanced ECL reagent followed with washing again. The relative levels of each protein normalized to *β*-actin were quantified with Image J software.

### 2.14. Statistical Analysis

Data were analyzed using SPSS version 16.0 statistical analysis software (SPSS Inc., Chicago, IL, USA). All data were represented as mean ± SD. The *P* value was calculated using one-way analysis of variance (ANOVA). *P* < 0.05 indicated statistical significance.

## 3. Results and Discussion

### 3.1. Results

#### 3.1.1. WXG Alleviated H/R-Induced H9c2 Cell Injury

**Figure 1:**
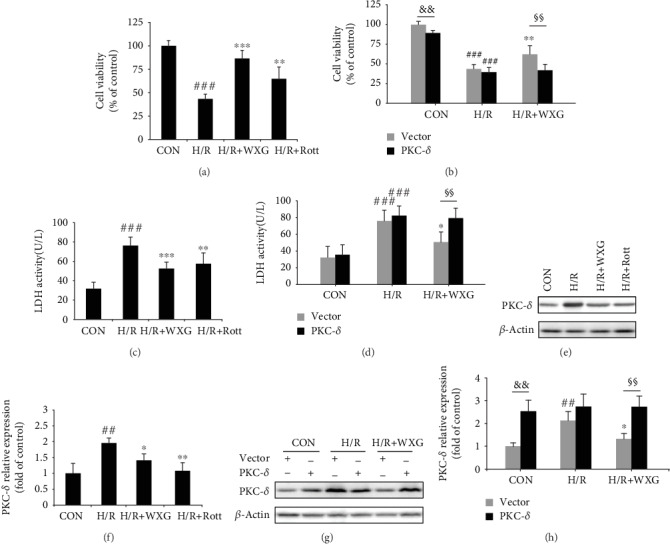
Overexpression of PKC-*δ* reversed the positive effect of WXG on H9c2 cells under H/R. (a, b) CCK-8 assay showed the proliferation of H9c2 cells under H/R (*n* = 6). (c, d) LDH release assay showed cell injury in each group (*n* = 6). (e–h) Western blot revealed PKC-*δ* protein expression (*n* = 3). Representative immunoblots were normalized to *β*-actin. ^##^*P* < 0.01, ^###^*P* < 0.001 vs. the control group; ^∗^*P* < 0.05, ^∗∗^*P* < 0.01, and ^∗∗∗^*P* < 0.001 vs. the H/R group. ^&&^*P* < 0.01, ^§§^*P* < 0.01.

After pretreatment with WXG (5 mg/kg) for 24 h, H9c2 cells were exposed to H/R. To evaluate the effect of PKC-*δ*, PKC-*δ* specific inhibitor rottlerin (Rott, 5 *μ*M) was added 1 h before H/R. To examine the effects of WXG and Rott, cell viability was measured using the CCK-8 assay kit. The results showed that cell viability dramatically decreased after H/R treatment. However, WXG and Rott treatments both significantly increased cell viability (Figure 1(a)). The release of LDH is widely used as a marker of cell membrane injury and complementary technique to assess cell viability after H/R. Our results showed that H9c2 cells, when suffered H/R, significantly increased cellular LDH leakage. When pretreated with WXG or Rott, there was a significant reduction in LDH release compared to the H/R group (Figure 1(c)). Western blotting showed that PKC-*δ* expression was significantly upregulated by H/R stimulation but downregulated after WXG or Rott treatment (Figures 1(e) and 1(f)). The results obtained suggested that WXG or Rott alleviated H/R injury in H9c2 cells.

To further investigate whether PKC-*δ* plays the key role in WXG treatment, we used lentivirus particles carrying PKC-*δ* to significantly overexpress PKC-*δ*. The markedly increased expression of PKC-*δ* (Lv-PKC-*δ*) compared with the empty vector group (Lv-CON) was confirmed by western blotting assay (Figures 1(g) and 1(h)). WXG had no significant impact on the protein level of PKC-*δ* in the H/R+WXG+Lv-PKC-*δ* group (Figures 1(g) and 1(h)). The results of CCK-8 assay showed that cell viability was dramatically decreased after H/R treatment (Figure 1(b)). However, the protective effect of WXG was reversed by PKC-*δ* overexpression. The release of LDH was consistent with the results of CCK-8 assay (Figure 1(d)). These data suggested the detrimental effects of PKC-*δ* overexpression on H9c2 cells under H/R and WXG treatment.

#### 3.1.2. WXG Attenuated H/R-Induced H9c2 Cell Oxidative Stress

**Figure 2:**
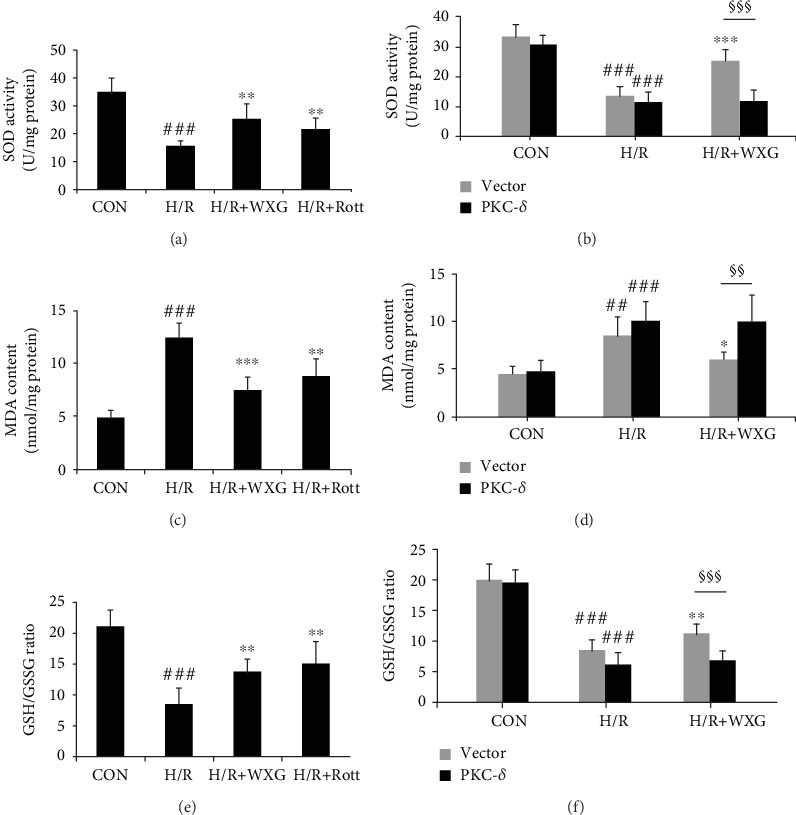
WXG rescued H/R-induced H9c2 cell oxidative stress injury which was reversed by PKC-*δ* overexpression. (a, b) SOD activity was determined by the SOD assay. (c, d) MDA content was estimated by the MDA assay. (e, f) GSH/GSSG ratios were estimated by the GSH and GSSG assay. *N* = 6 in each group. ^##^*P* < 0.01, ^###^*P* < 0.001 vs. the control group; ^∗^*P* < 0.05, ^∗∗^*P* < 0.01, and ^∗∗∗^*P* < 0.001 vs. the H/R group. ^§§^*P* < 0.01, ^§§§^*P* < 0.001.

To investigate the effects of WXG on H/R-induced oxidative stress in H9c2 cells, we examined the biochemical markers of oxidative stress, including SOD activity, MDA level, and GSH/GSSG ratio. As presented in Figures 2(a), 2(c), and 2(e), oxidative stress injury was induced by H/R treatment, as indicated by the dramatically upregulation of MDA levels and the significant decrease of SOD activity and GSH/GSSG ratio, compared with the control group. Pretreatment of WXG or Rott significantly increased the SOD activity and the GSH/GSSG ratio of H/R-treated H9c2 cells. Meanwhile, the levels of MDA were significantly decreased in the WXG group compared with those in the H/R group. In contrast, the improvement of SOD activity, MDA level, and GSH/GSSG ratio was suppressed by PKC-*δ* overexpression (Figures 2(b), 2(d), and 2(f)). All the above results demonstrated that WXG could markedly alleviate H/R-induced oxidative abnormalities and restore endogenous antioxidant systems in H9c2 cells, whereas the antioxidative activity was attenuated by PKC-*δ* overexpression.

#### 3.1.3. WXG Mitigated H/R-Induced ROS Overproduction

**Figure 3:**
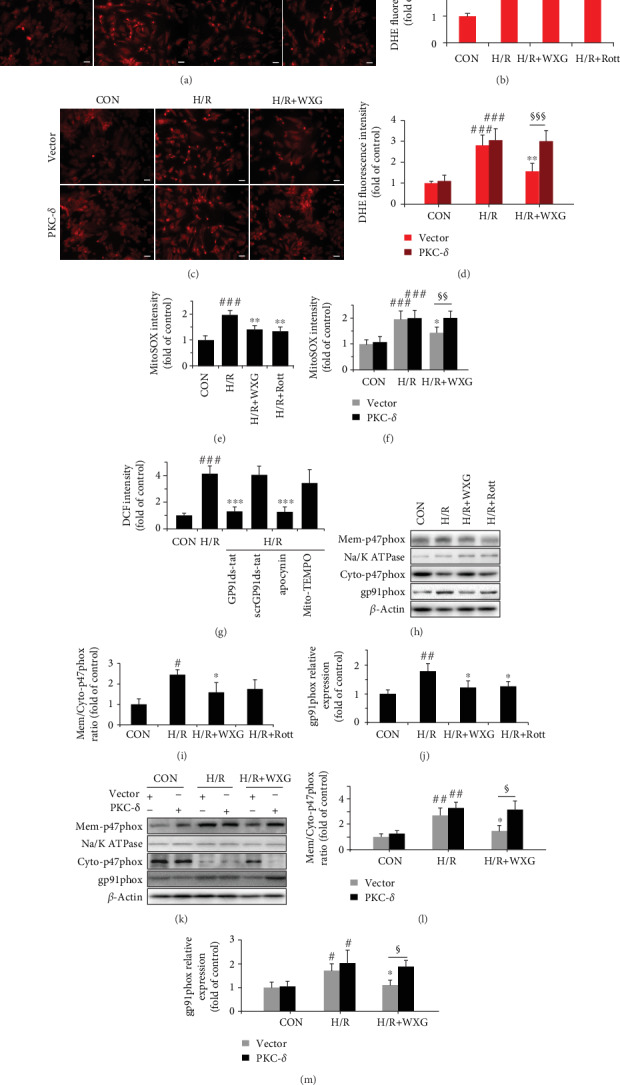
WXG suppressed ROS generation in H/R-induced H9c2 cells which was reversed by PKC-*δ* overexpression. (a–d) Representative fluorescence images (left panel) and quantification (right panel) of DHE fluorescence (*n* = 6). Sale bar =20 *μ*M. (e, f) Quantification of MitoSOX fluorescence in each group (*n* = 6). (g) Quantification of DCF fluorescence in each group (*n* = 6). (h–m) Western blot revealed p47phxo (membrane/cytosolic) and gp91phox protein expression (*n* = 3). Representative immunoblots were normalized to *β*-actin. ^#^*P* < 0.05, ^##^*P* < 0.01, and ^###^*P* < 0.001 vs. the control group; ^∗^*P* < 0.05, ^∗∗^*P* < 0.01 vs. the H/R group. ^§^*P* < 0.05, ^§§^*P* < 0.01, and ^§§§^*P* < 0.001.

Our previous studies showed WXG resulted in intracellular oxidative stress improvement in H/R-induced H9c2 cells. Here, to provide more direct evidence, we detected the generation of intracellular ROS and mitochondrial ROS by using DHE and MitoSOX staining, respectively. H/R-induced intracellular ROS levels were detected by using DHE, and results are represented in Figures 3(a) and 3(b). The levels of intracellular ROS were clearly increased in the H/R group compared to the control group, and these increases were significantly reduced by WXG or Rott. Importantly, overexpressed PKC-*δ* could markedly prevent the WXG-induced intracellular ROS downregulation (Figures 3(c) and 3(d)). On the other hand, to intensify subcellular localization specificity, mitochondrial ROS generation in H9c2 cells subjected to H/R was measured by flow cytometry with MitoSox. H/R exposure significantly elevated mitochondrial ROS levels in H9c2 cells, which were reversed by WXG pretreatment (Figure 3(e)). Moreover, consistent with the results of intracellular ROS, WXG exhibited not obvious effects on mitochondrial ROS levels when PKC-*δ* overexpressed in H9c2 cells (Figure 3(f)). To identify the ROS source, we pretreated H9c2 cells with NOX2 inhibitor GP91ds-tat, NOX inhibitor apocynin, and mitochondrial ROS scavenger Mito-TEMPO. DCF fluorescence intensity showed the full ROS inhibition by GP91ds-tat and apocynin and lack of inhibition by scrGP91ds-tat and Mito-TEMPO (Figure 3(g)). These results suggested that H/R injury mainly triggered NOX2-related ROS, and mitochondrial ROS might be induced by increased intracellular oxidative stress. Western blotting assay showed that p47phox translocated from the cytoplasm to the cytomembrane accompanied with the upregulation of gp91, which further confirmed the activation of NOX2 after H/R stimulation (Figures 3(h)–3(j)). WXG significantly prevented the translocation of p47phox and the activation of NOX2. However, the overexpression of PKC-*δ* abolished the regulatory effects of WXG in H/R-treated H9c2 cells (Figures 3(k)–3(m)). These data implied that H/R caused oxidative stress in H9c2 cells and the antioxidant potential of WXG; therefore, we hypothesize that PKC-*δ* may be an essential role involved in WXG-mediated antioxidative activity.

#### 3.1.4. WXG Moderated H/R-Induced Dissipation of Mitochondrial Membrane Potential and ATP

**Figure 4:**
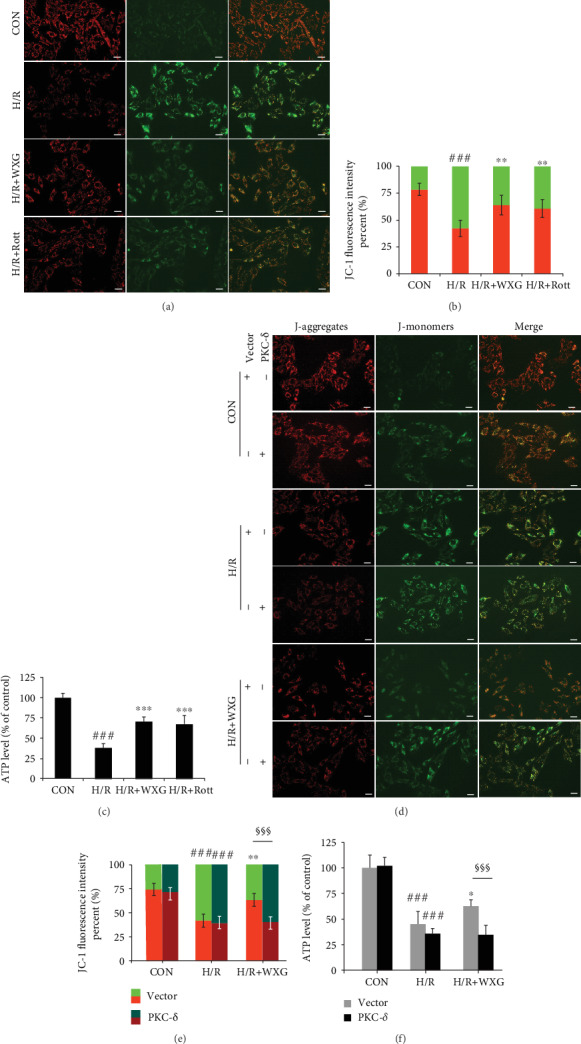
WXG inhibited the collapse of mitochondrial transmembrane potential and ATP level in H/R-induced H9c2 cells which was reversed by PKC-*δ* overexpression. (a, d) Representative fluorescence images of JC-1 fluorescence. Scale bar =20 *μ*M. (b, e) Quantification (right panel) of JC-1 fluorescence (*n* = 6). (c, f) ATP level was assessed using an ATP detection kit (*n* = 6). ^###^*P* < 0.001 vs. the control group; ^∗^*P* < 0.05, ^∗∗^*P* < 0.01 vs. the H/R group. ^§§§^*P* < 0.001.

The mitochondrial *ΔΨ*m and intracellular ATP level are valuable indicators of mitochondrial function and the apoptotic status of a cell, and loss of *ΔΨ*m and ATP level indicates mitochondrial dysfunction and cell apoptosis. To evaluate the protective effect of WXG on H/R-induced mitochondrial membrane damage, *ΔΨ*m and ATP level were assessed, respectively, to evaluate mitochondrial function in H9c2 cells. Figures 4(a) and 4(b) show that in normal H9c2 cells, JC-1 probe was mainly in the aggregated state resulting in a higher J-aggregate (red fluorescence)/J-monomer (green fluorescence) ratio, suggesting a consequence of a higher membrane potential. When exposed to H/R, H9c2 cells exhibited a complete loss of *ΔΨ*m and ATP level (Figure 4(c)). Pretreatment with WXG or Rott attenuated *ΔΨ*m and ATP depletion, and these effects were abolished when PKC-*δ* overexpressed (H/R+WXG+Lv-PKC-*δ* group) (Figures 4(d)–4(f)). These results clearly indicated that WXG moderated the dissipation of mitochondrial transmembrane potential and ATP level in H9c2 cells induced by H/R. In contrast, overexpression of PKC-*δ* prevented the protective effect of WXG in the mitochondria which was clearly evident from the loss of *ΔΨ*m and ATP level, and the feeble *ΔΨ*m implied the primary change of cell apoptosis.

#### 3.1.5. WXG Alleviated H/R-Induced Mitochondrial Permeability Transition Pore Opening

**Figure 5:**
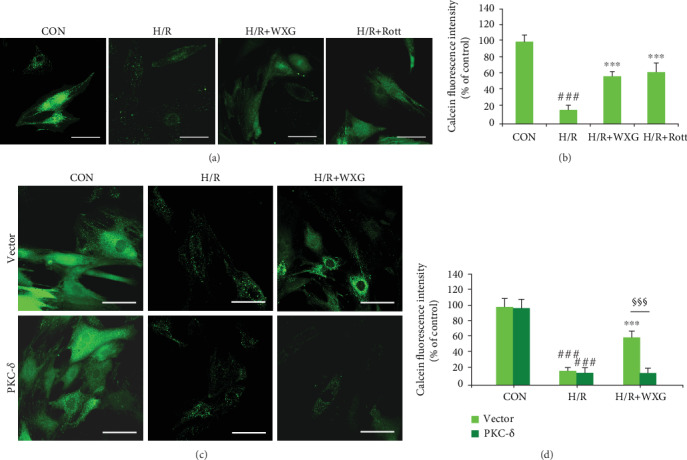
WXG decreased opening of the mitochondrial permeability transition pore in H/R-induced H9c2 cells which was reversed by PKC-*δ* overexpression. (a–d) Representative fluorescence images and quantification of calcein fluorescence (*n* = 6). Scale bar =50 *μ*M. ^###^*P* < 0.001 vs. the control group; ^∗∗∗^*P* < 0.001 vs. the H/R group. ^§§§^*P* < 0.001. (e–h) Western blot revealed cytochrome C protein expression (*n* = 3). Representative immunoblots were normalized to *β*-actin. ^##^*P* < 0.01, ^###^*P* < 0.001 vs. the control group; ^∗^*P* < 0.05, ^∗∗^*P* < 0.01, and ^∗∗∗^*P* < 0.001 vs. the H/R group. ^§^*P* < 0.05, ^§§§^*P* < 0.001.

MPTP is an indicator of mitochondrial function, and alterations of mitochondrial function have been considered vital in cell apoptosis. The modification of mPTP open/closed status is an early event in the induction of apoptosis. The occurrence of mPTP opening was investigated by detecting the green fluorescence of mitochondrial-entrapped calcein. As shown in Figures 5(a) and 5(b), compared with the control group, the calcein fluorescence was markedly reduced in the H/R group, indicating mPTP activation. The pretreatment of WXG or Rott significantly mitigated H/R-induced mPTP opening as shown in increased green fluorescence. To determine whether PKC-*δ* was related to mPTP changes, H9c2 cells were transfected with overexpressed PKC-*δ*. PKC-*δ* overexpressing markedly decreased the fluorescence intensity compared with the WXG-treated group under H/R stimulus (Figures 5(c) and 5(d)). Thus, PKC-*δ* was highly related to WXG-induced mitochondrial protection in the maintenance of mPTP. These findings were consistent with the *ΔΨ*m measurement, previously.

**Figure 6:**
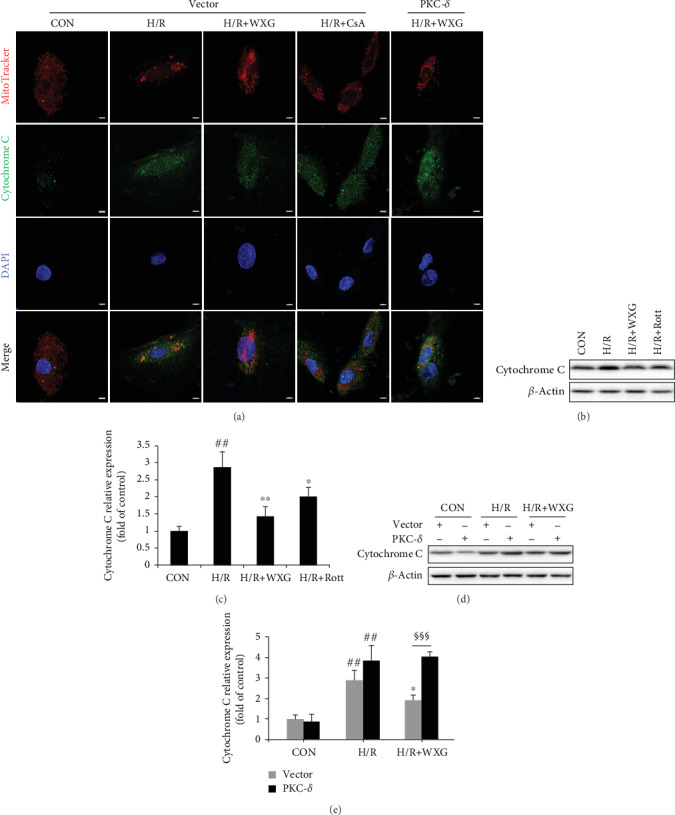
WXG decreased cytochrome C increase and release in H/R-induced H9c2 cells which was reversed by PKC-*δ* overexpression. (a) Representative fluorescence images of MitoTracker and cytochrome C fluorescence (*n* = 6). Scale bar = 5 *μ*M. (b–e) Western blot revealed cytochrome C protein expression (*n* = 3). Representative immunoblots were normalized to *β*-actin. ^##^*P* < 0.01, ^###^*P* < 0.001 vs. the control group; ^∗^*P* < 0.05, ^∗∗^*P* < 0.01 vs. the H/R group. ^§^*P* < 0.05, ^§§§^*P* < 0.001.

The mPTP opening was associated with a collapse of *ΔΨ*m and cytochrome C release, which resulted in a vicious cycle of ROS production and mitochondrial dysfunction. To further examine the mPTP opening, cytochrome C release from mitochondria was detected by MitoTracker and cytochrome C immunofluorescence staining using confocal microscopy, and the total cytochrome C protein level was measured by western blotting assay. Data showed that H/R treatment increased cytochrome C release (Figure 6(a)) and total protein level (Figures 6(b) and 6(e)) of cytochrome C compared with normal H9c2 cells. WXG pretreatment did prevent cytochrome C release and upregulation in H9c2 cells; however, in PKC-*δ* overexpressed H9c2 cells, the prevention of cytochrome C release and upregulation was partially reversed. Furthermore, mPTP inhibitor cyclosporine A partially decreased cytochrome C release (Figure 6(a)). Collectively, these results demonstrated that H/R triggered a PKC-*δ*-involved opening of the mPTP that likely accompanied cytochrome C release and upregulation, which could be alleviated by WXG.

#### 3.1.6. WXG Attenuated H/R-Induced H9c2 Cell Apoptosis

**Figure 7:**
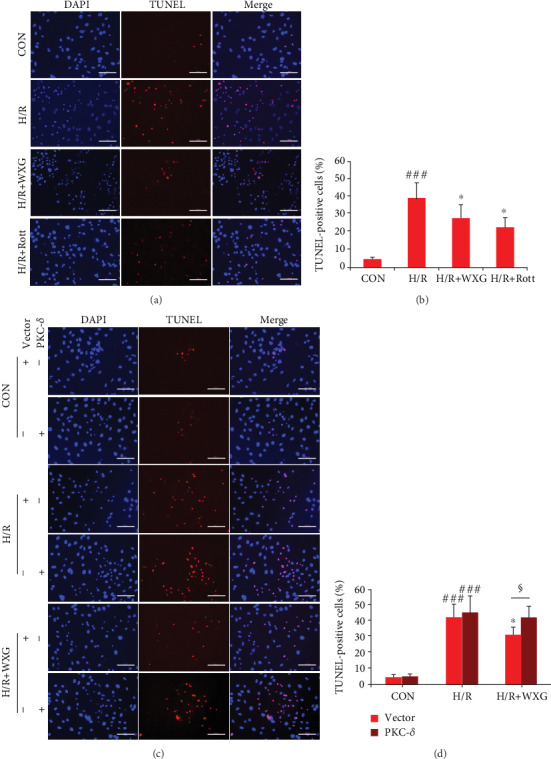
WXG reduced H/R-triggered apoptosis in H9c2 cells which was reversed by PKC-*δ* overexpression. (a–d) Representative fluorescence images of TUNEL (green) and DAPI (blue) and quantification of TUNEL-positive cells (*n* = 6). Scale bar = 100 *μ*M. ^###^*P* < 0.001 vs. the control group; ^∗^*P* < 0.05 vs. the H/R group. ^§^*P* < 0.05.

**Figure 8:**
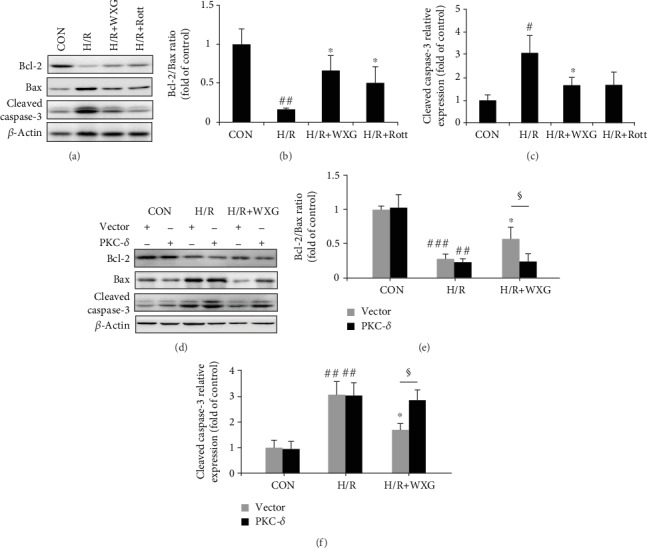
WXG downregulated apoptosis protein in H/R-induced H9c2 cells which was reversed by PKC-*δ* overexpression. (a–f) Western blot revealed Bcl-2, Bax, and cleaved caspase-3 protein expression (*n* = 3). Representative immunoblots were normalized to *β*-actin. ^#^*P* < 0.05, ^##^*P* < 0.01, and ^###^*P* < 0.001 vs. the control group; ^∗^*P* < 0.05 vs. the H/R group. ^§^*P* < 0.05.

The TUNEL reaction is well-known for cell apoptosis. H/R stimulus significantly increased the proportion of TUNEL-positive cells compared with normal H9c2 cells (Figures 7(a) and 7(b)). Pretreatment with WXG or Rott significantly reduced the apoptotic index compared to the H/R group. Bcl-2 and Bax, as antiapoptotic protein and proapoptotic protein, both played crucial roles in regulating cell apoptosis. In the H/R group, the protein expression of Bcl-2 was markedly lower compared to control H9c2 cells, while the protein levels of Bax and cleaved caspase-3 were significantly higher, and all of which were ameliorated by pretreatment with WXG or Rott (Figures 8(a)–8(c)). These results were consistent with the TUNEL assay. Furthermore, the effects of WXG on H9c2 cell apoptosis were blocked by PKC-*δ* overexpression (Figures 7(c) and 7(d), Figures 8(d)–8(f)). These data further supported the hypothesis that the PKC-*δ* played an essential role in the antiapoptosis mechanism of WXG.

## 4. Discussion

WXG is a clinically used Chinese patent medicine for the treatment of cardiovascular diseases, such as arrhythmia and heart failure [40]. In addition, WXG experimentally restores a large number of myocardial infarction and ischemia/reperfusion injuries [29, 31]. Whether WXG has some additional beneficial effects on cardiomyocytes following I/R injury requires further study. H9c2 cells were subjected to hypoxia for 16 h and reoxygenation for 2 h to mimic the I/R injury *in vitro*, namely, the H/R group. We demonstrated that H/R treatment significantly decreased cell viability and increased LDH release compared to the control group. After pretreated 24 h with 5 mg/mL WXG, H9c2 cells showed dramatically improved cell viability and LDH leakage. The above findings confirmed that WXG could rescue H/R-induced H9c2 cell injury.

Signaling proteins like protein kinase C (PKC), protein kinase A (PKA), phospholipase A2 (PLA2), phospholipase D, mitogen-activated protein kinases (MAPK), and phosphatidylinositol-3-kinase (PI3K) are clearly defined NOX activators [41, 42]. And, PKC, especially the *δ* form, is known to be involved in NOX2-derived ROS generation and subsequent myocardial oxidative stress [43, 44]. Previously, we investigated that H/R stimuli significantly increased PKC-*δ* expression, and WXG pretreatment significantly downregulated the protein level of PKC-*δ* compared to the H/R group. Although the molecular mechanism of PKC-*δ*-mediated redox-dependent signaling remains incompletely understood, there is growing evidence indicating that PKC-*δ* participates in the oxidative stress process in different cell types [45, 46]. In the present study, we addressed the role of PKC-*δ* in mediating WXG antioxidative activity under H/R through the regulation of the NOX2/ROS signaling pathway. To further identify whether the existence of PKC-*δ* was critical in this mechanism, we used PKC-*δ* inhibitor Rott or PKC-*δ* overexpressing H9c2 cells. In this study, we evaluated the expression of PKC-*δ* in different groups. Rott significantly decreased the upregulation of PKC-*δ* induced by H/R in H9c2 cells. Moreover, in PKC-*δ* overexpressing H9c2 cells, WXG showed no obvious efficacy on PKC-*δ* expression.

PKC can lead to NOX2 activation with complicated interactions with NOX2 subunits, including the activation and translocation of cytosolic p47phox to the membrane [47]. Then, the p47phox binds and activates membrane p22phox and gp91 (as the catalytic core of NOX) heterodimer [48]. With such localization and activation, the NOX2/ROS signaling pathway is initiated. In the H/R group, we observed that p47phox translocated from cytosol to cytomembrane with markedly increased gp91 expression compared with normal H9c2 cells. WXG or Rott significantly inhibited the relocation of p47phox and the increase of gp91. NOX2 and NOX inhibitors, mitochondrial ROS scavenger, were used to confirm that the main source of ROS was NOX2. Consistent with the activation of NOX2 as the major source of ROS in H/R-induced oxidative stress in H9c2 cells, two independent ROS detection techniques (DHE fluorescence and MitoSOX) demonstrated that H/R-induced ROS production was significantly inhibited by WXG or Rott compared to controls. However, overexpression of PKC-*δ* augmented the translocation of p47phox and increase of gp91 and seriously accumulated intracellular and mitochondrial ROS. The cellular oxidative injury degree (SOD activity, MDA level, and GSH/GSSG ratio) further confirmed the previous results. These findings demonstrated that overexpression of PKC-*δ* in H9c2 cells abolished WXG-alleviated oxidative stress in H9c2 cells in response to H/R treatments. These results provided a mechanistic linking between the PKC-*δ* protein level and p47phox relocalization to induce NOX2 activation-mediated ROS overproduction and redox imbalance under H/R and WXG treatments. These results also suggested that PKC-*δ* represented a potential target for WXG ameliorating H/R-induced oxidative stress in H9c2 cells.

Although there are several sources of ROS in cardiac I/R injury, an increasing amount of evidence has shown that the main ROS source during myocardial I/R appears to be NOX2 in mitochondria [18, 49]. Mitochondria are the center of energy and metabolism of eukaryotes, which play pivotal roles in numerous homeostatic processes, including energy generation, calcium homeostasis, redox balance, cell death programs, and cell senescence [50]. Mitochondria are abundantly expressed in the myocardium as an energy supporter for myocardial contraction [51, 52]. Mitochondrial homeostasis is considered a vital target in cardiac I/R therapy, due to their insufficient supply of energy, excessive generation of ROS, and releasing cytochrome C and other proapoptotic factors [53, 54]. We investigated whether PKC-*δ*/NOX2-derived ROS contributed to the stimulation of mitochondrial dysfunction in response to H/R treatments in H9c2 cells. Both WXG and Rott significantly attenuated mitochondrial membrane potential depletion, ATP loss, and mPTP opening induced by H/R stimuli, as well as the cytosol release of cytochrome C. TUNEL assay and western blot analysis also revealed that the apoptotic index, indicated by TUNEL-positive cells, the Bcl-2/Bax ratio, and cleaved caspase-3 level, were also significantly reduced by WXG or Rott pretreatment compared to the H/R group. However, the protective effects of WXG on H/R-induced mitochondrial injury and apoptosis were reversed by PKC-*δ* overexpression. These data indicated the key role of PKC-*δ* as a signaling molecule in WXG-mediated H9c2 cell protection stimulated by H/R.

## 5. Conclusions

**Figure 9:**
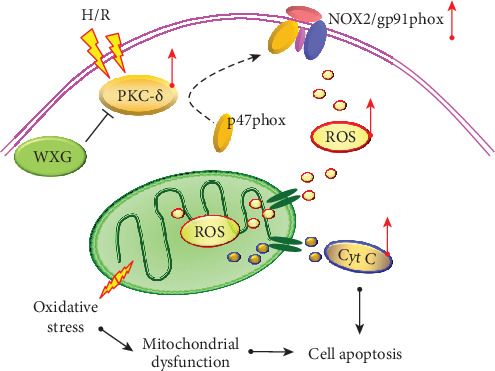
WXG rescued H9c2 cells under H/R stimulation by attenuating mitochondrial oxidative stress via PKC-*δ*/NOX2/ROS inactivation.

In this work, we described a novel mechanism for mitochondrial protection via PKC-*δ*/NOX2/ROS inactivation induced by WXG in H9c2 cells after H/R stimulation which mimicked the myocardial I/R injury model. Exploration of underlying mechanisms revealed that WXG alleviated cellular oxidative stress and ultimate apoptosis, by suppressing PKC-*δ* upregulation, p47phox cytomembrane translocation, gp91 increase, NOX2 activation, ROS overproduction and accumulation, and apoptotic factor activation and release (Figure 9). We demonstrated that overexpression of PKC-*δ* abolished the protective effects of WXG against H/R-induced cell injury. Finally, our findings pointed to a necessary role for PKC-*δ*-dependent activation of the NOX2/ROS signaling pathway in the antioxidative effects of WXG under H/R.

## Data Availability

The data that have been used in this research are available from the corresponding author upon request.
